# Clinician attitudes towards adoption of evidence-based practice: a nationwide multiprofessional cross-sectional study of child and adolescent mental health services in Sweden

**DOI:** 10.1186/s12913-024-11934-9

**Published:** 2024-11-19

**Authors:** Anna Helena Elisabeth Santesson, Robert Holmberg, Martin Bäckström, Peik Gustafsson, Håkan Jarbin, Sean Perrin

**Affiliations:** 1 Department of Clinical Sciences, Faculty of Medicine, Lund University, BMC F12, S- 221 84 Lund, Sweden; 2 Department of Psychology, Faculty of Social Sciences, Lund University, Box 213, S-221 00 Lund, Sweden; 3 Child and Adolescent Psychiatry, Region Halland, S- 30185 Halland, Sweden

**Keywords:** Evidence-Based Practice (EBP), Attitudes, Evidence-Based Practice Attitude Scale (EBPAS), Implementation, Mental health, Child-and Adolescent Mental Health Service (CAMHS)

## Abstract

**Background:**

Implementation of evidence-based practice (EBP) in child and adolescent mental health services (CAMHS) is a priority to improve service delivery and outcomes. Clinicians’ EBP attitudes are likely to play a crucial role in implementation but are poorly understood. This study aimed to assess variation in EBP attitudes in a large national sample of CAMHS clinicians in Sweden, and to compare these findings to findings from the United States of America (USA).

**Methods:**

CAMHS clinicians (*n* = 799; 60% response rate) completed the Evidence-Based Practice Attitude Scale (EBPAS) and items from the Organizational Readiness for Change Scale (ORC) ahead of an EBP for depression implementation effort across Sweden. EBPAS scores were compared with the USA study. Predictors of global and specific attitudes (gender, age, working years, education, profession, perceived benefit of diagnosis and organizational readiness and type of service) were examined using simple and multiple linear regressions.

**Results:**

Clinicians had positive attitudes towards EBP on the four-dimensional subscales of the EBPAS, somewhat more so than their American counterparts. Clinician and organizational characteristics were related to at least one attitudinal dimension in both models, with perceived utility of diagnosis being the strongest and most consistent predictor across dimensions and models.

**Conclusions:**

Results from this large-scale national study underscore the need to consider cultural, contextual, and individual variations in attitudes towards EBP when planning implementation efforts. Such efforts may need to be tailored to the working contexts, needs, and values of CAMHS clinicians, particularly their views on the utility of diagnosis.

**Supplementary Information:**

The online version contains supplementary material available at 10.1186/s12913-024-11934-9.

## Introduction

Despite years of research on their development and testing, empirically supported methods of assessment and treatment are not reaching enough of the youth seen in child and adolescent mental health services (CAMHS) across countries [1–3]. Consequently, governments, policymakers and healthcare providers have re-prioritized their efforts to disseminate, and improve the uptake of, empirically supported methods in CAMHS [4, 5]. Such efforts can be seen as part of a larger effort across child and adult mental health to improve clinical outcomes at the local level by helping clinicians to improve their decision making and practice by integrating the latest scientific findings, often summarized in national or local care guidelines, with the needs and values of their patients; referred to as Evidence-Based Practice (EBP) [6]. EBP originates from Evidence-Based Medicine and involves ‘the conscientious, explicit, and judicious use of current best evidence in making decisions about the care of individual patients’ [6]. The definition of EBP was adopted and adapted by the Institute of Medicine and further refined to ‘the integration of the best available research with clinical expertise in the context of patient characteristics, culture, and preferences’ in the context of psychology [7–9]. EBP is a comprehensive concept that encompasses, but is not limited to, the use of empirically supported treatments (ESTs) or assessments. An accurate diagnosis is crucial for clinical decision-making and may act as a facilitator to receive appropriate ESTs [10]. However, it may also act as a barrier due to the complexity of cases in ordinary practice and the corresponding difficulty and time lag in obtaining an accurate diagnosis [10]. The usefulness of diagnosis and diagnostic aids for treatment selection and prognosis in ordinary clinical practice has therefore been questioned by practicing clinicians [11]. How perceptions of diagnosis utility relate to attitudes towards EBP remains unclear.

A key factor in the uptake of EBPs at the local level, is the readiness of the clinicians and organization to adopt “new” practices [12–14]. Clinicians are particularly important stakeholders, as their attitudes towards EBP broadly, and the adoption of particular methods, will influence their willingness to adopt new ways of working with patients [15, 16]. There is a small but growing body of evidence suggesting that positive EBP attitudes are significantly related to EBP adoption, but varying along individual (age, gender, educational attainment, experience), organizational (leadership, resources, levels of stress, support, service type), and patient characteristics (age, diagnosis, complexity) [5, 17, 18]. However, more research is needed to understand the interplay between these factors [5].

The Evidence-Based Attitude Practice Scale (EBPAS) is a widely used, 15-item measure of clinician’s EBP attitudes along four dimensions: the intuitive appeal of EBP; the likelihood of adopting EBP given requirements to do so; openness to new practices; and perceived divergence between research-based/academically developed interventions and current practice [13]. The scale has been shown to have satisfactory validity and reliability [13, 19–25]. Importantly, total and subscale scores have been found to be related to initial adoption, fidelity, and sustained use of EBP in mental health settings [5, 13, 26, 27]. National norms are available for the United States of America (USA) that can be used for benchmarking across countries [19, 21, 22].

Clinicians working in mental health settings are heterogenous in relation to background, roles, disciplines, positions, and workplace characteristics, all of which may influence their EBP attitudes [28]. Studies employing the EBPAS have found that women, younger, and less experienced, but more highly educated mental health providers tend to report more favourable EBP attitudes, although results are somewhat inconsistent across studies [13, 14, 19, 23, 24, 29]. The clinician’s discipline may be expected to influence EBP attitudes, as some disciplines place greater emphasis on combining research and practice during training and post-qualification [13, 19, 28]. Such variation has been found with social workers reporting more positive EBP attitudes [13, 14]. Differences between disciplines outside the USA remains poorly understood owing to few studies, sampling procedures or small sample sizes [23, 24, 30, 31].

Implementation frameworks suggest a complex interplay between organizational and individual implementation determinants [32]. More positive EBP attitudes are found in individuals working in more proficient, engaged, supportive and less stressful work environments, but varying between public vs. private, academic vs non-academic organizations and leadership style [12, 14, 30, 33, 34]. It is likely that organizational factors impact on clinician EBP use, interact with clinician characteristics, including knowledge of and attitudes towards EBP, with more research needed on this topic [35–37].

The EBPAS has been used in a variety of settings, countries and cultures, most notably within the area of behavioural health, but no study outside the USA has surveyed a nationally representative sample [14, 23, 24, 30]. This includes Sweden, where no study has examined EBP attitudes in clinicians working in CAMHS. Results from a Norwegian study found significant differences in EBP attitudes, showing more positive attitudes toward EBP adoption when it was appealing, greater openness to innovation, and less divergence, when compared with normative data from the USA [23]. Given the similarities between Norway and Sweden in terms of the healthcare training and delivery, similar differences may exist between Sweden and the USA for EBP attitudes in CAMHS clinicians. Partly consistent with such a view, a Swedish study of 345 clinicians working in inpatient and outpatient CAMHS in Stockholm were more positively disposed towards standardized assessments and diagnosis than normative data from the USA using the same questionnaires [38]. The authors also found a good deal of variability in attitudes towards assessment based on clinician and organizational characteristics. These studies utilized t-tests for comparison, even without direct access to the original data, as this approach is widely accepted for benchmarking normative data across different populations in this field.

In summary, there is preliminary evidence that clinicians’ attitudes towards EBP are a key factor in the success of EBP implementation efforts. These attitudes appear to vary according to clinician and workplace characteristics, but firm conclusions are limited by the number of studies and sampling procedure and sample size issues. The present study aimed to address a gap in the literature with respect to EBP attitudes among clinicians working in routine CAMHS in Sweden. To address some of the methodological limitations of previous studies, the EBPAS was administered to a nationally representative sample of CAMHS clinicians in Sweden and their responses were compared to normative data from the USA. Based on the available literature, we hypothesized that: a) CAMHS clinicians would be positive towards EBP; b) would be more positive compared to normative data for the EBPAS from the USA; c) EBPAS scale scores would vary by sex, age, educational attainment, experience, profession, attitude toward diagnosis, organizational readiness, and service setting; and d) some of these background and organizational factors would remain significant predictors of EBP attitudes when controlling for sex, age, educational attainment, experience attitude to diagnosis and organizational readiness, and service setting.

## Methods

### Design and setting

Data from the present cross-sectional study was collected at baseline in a large multi-CAMHS implementation study of child and adolescent depression guidelines in Sweden [25, 39, 40]. All Swedish publicly (state) funded CAMHS were invited and 16 of 31 eligible CAMHS. These participating CAMHS serve about 66% of Swedish youth participate, covering a similar-sized catchment area and approximately 62,000 (25,000–250,000) children, as compared to the remaining CAMHS, which serve about 64,000 (29,000–450,000) children. A web-based survey was administered to 1350 outpatient CAMHS clinicians from October 2014 to June 2018. Two to five reminders were sent, and no compensation was offered to the clinicians. The survey included questions about the clinician’s age, gender, professional discipline, highest educational level, number of years worked in CAMHS, followed by the EBPAS, questions from the Organizational Readiness for Change (ORC) and a single question about the usefulness of psychiatric diagnosis [13, 41]. This single not previously used question about the usefulness of psychiatric diagnosis (Likert scale 1–5 with an additional “Not applicable option”) was developed specifically for the purpose of this study. (Supplemental table S3).

### Participants

A total of 812 clinicians completed the survey (a 60% response rate). Of these, twelve were excluded because of missing all items on the EBPAS and one because all responses were the same, leaving 799 participants (Table 1). Missing data was less than 5% for demographic data (Table 1), EBPAS items (Supplemental table S1), and items from the Organizational Readiness for Change (ORC) [13, 41] (Supplemental table S2) and the item about utility of diagnosis (S3). The typical participant was female (84%), 35–45 years old (28%), a psychologist (33%) with less than 5 years’ experience of child and adolescent psychiatry (44%) (Table 1).

**Table 1:** Background characteristics of respondents (*n* = 799)

	n	%
Gender		
Male	128	16.2
Female	660	83.8
Age Group		
< 35 years	167	21.2
35–44 years	216	27.4
45–55 years	196	24.9
> 55 years	209	26.6
Education		
< University	21	2.7
Bachelor	521	65.8
Master	220	27.8
PhD	30	3.8
Profession		
Auxiliary nurse	27	3.4
Nurse	11	13.9
Social worker	207	26.0
Psychologist	263	33.0
Psychiatrist*	104	13.0
Other	85	10.7
Tenure Child Mental health		
< 5 years	345	43.7
5–10 years	138	17.5
11–15 years	98	12.4
16–20 years	82	10.4
> 20 years	126	16.0
Type of workplace /Service		
Non academic	489	61.2
Academic	310	38.8

Sample sizes vary slightly because of missing data. < University refers to secondary school (mandatory to age 16) or gymnasium (age 16–19 years)

^*^Psychiatrists include Child Psychiatrists, residents, and MDs without any specialist training

### Measure/s

#### EBP Attitudes

EBP attitudes were measured with the Swedish version of the 15-item EBPAS [25]. Items are rated on a 5-point scale ranging from 0 (not at all) to 4 (to a very great extent) with four subscales measuring: 1) the intuitive appeal of EBP (Appeal, four items); 2) the likelihood of adopting EBPs given requirements to do so (Requirements, three items); 3) openness to new or more structured practices (Openness, four items); and 4) perceived clinical usefulness of and divergence between research-based developed interventions and current practice (Divergence, four items, reverse scored) [13]. Subscale means and a total scale score are computed, with higher total and subscale means indicating more positive EBP attitudes and less divergence between EBP and current practice. Previous studies report adequate internal consistency for the English language original [13, 19, 20]. Psychometric properties of the Swedish version were on par with the English language original [25]. In the present sample, the internal consistency values were as follows: EBPAS total scale α = 0.83; Requirements α = 0.89; Appeal α = 0.78; Openness α = 0.78; and Divergence α = 0.63.

#### Organizational readiness

Items from the Organizational Readiness for Change (ORC) [41] were used to assess clinicians’ perceptions of organizational readiness. The ORC is comprised of 115 items scored on a 5-point scale (1 = strongly disagree, 5 = strongly agree), representing 18 content domains of organizational readiness for implementation. The ORC includes four subscales measuring motivational factors, program resources, and organizational climate at the organizational level and staff attributes at the individual practitioner level [41]. To reduce the item load of the overall survey, participants completed nine ORC items assessing organizational readiness: one item from each of the six subscales of the Organizational Climate scale, two items from the Motivation for Change scale; and one item from the Resources scale (staff turnover). To ensure that the most representative and relevant aspects of the subscales’ constructs were included, items were selected through a consensus procedure based on their content and the strongest factor loadings in a validated Swedish-language version was used in this study [42]. See supplemental table S2 for individual items. Internal consistency for the nine ORC items used in the present study was α = 0.71.

### Data analysis

The reporting of results was guided by the Standards for reporting Implementation studies (STaRI) and the Strengthening the Reporting of Observational Studies in Epidemiology (STROBE) statements respectively [43, 44]. All statistical analyses were carried out using Version 27 of SPSS [45]. We applied a Bonferroni correction to all analyses to control for Type 1 error given multiple comparisons. Prior to analyses, we examined the accuracy of data entry, missing values, normality, nonlinearity, and heteroscedasticity and influential cases for grouped and ungrouped data. We used independent sample t-tests to compare means on the four subscales of the EBPAS to USA norms. To estimate the magnitude of observed relationships, we used Cohen’s *d* with *d* = 0.20 corresponding to small, *d* = 0.50 to medium, and *d* = 0.80 to large effect sizes [46]. Intra class coefficients (ICC’s) were used to estimate the variance in EPBAS and ORC scores that could be attributed to individual CAMHS. All ICCs were low indicating that EBPAS and ORC scores should represent clinician-level and not CAMHS-level constructs. The highest ICCs were 0.021 for the Requirement subscale of the EBPAS and 0.06 for the nine-items from the ORC used in this study.

We conducted simple regressions, with listwise deletion, using the EBPAS scales as the dependent variables (DVs) and the following independent variables (IVs) for all regressions: gender, age, experience, highest level of education, profession, attitude towards psychiatric diagnosis, type of workplace (academic-non-academic), and organizational readiness (total score on the 9-item ORC). Next, we conducted multiple regressions using the same IVs and DVs. Since the ICCs were low, we replicated Aarons et al. (2004, 2010) multiple regression analyses, in line with the Norwegian and Dutch studies of the EBPAS [13, 19, 23, 24]. For the multiple regression analyses, squared semipartial correlation was used to estimate the unique relationship between IVs and DVs. These squared semipartial correlations and R2 from simple regressions enable comparison of effect sizes for each predictor, while the beta coefficients assist comparison of the strengths of predictors across models. The following benchmarks were used to estimate the strengths of the regression coefficients: *R*^2^ = 0.02—small: *R*^2^ = 0.13 – medium, and *R*^2^ = 0.26 – large [46].

### Data preparation

Three univariate outliers (*z* scores > 3.0) on the EBPAS and ORC scales were replaced with one unit less extreme [47]. No influential cases (Cook’s distance < 1) were found. The following categorical predictors with three or more categories were dummy coded: age (< 45 years vs ≥ 45 years), educational attainment (bachelor’s degree and lower vs master’s degree and higher), and clinical experience (< 5 years vs ≥ 5 years). Scale scores were the means of test items, provided at least 50% of scale items had valid data.

## Results

### Clinicians’ attitudes toward EBP adoption

Table 2 presents the means and standard deviations for the total and subscale scores on the EBPAS for the present sample and norms from the USA, t-test comparisons, and effect sizes. Overall, participants in this study expressed favourable EBP attitudes. Mean values were high for most of the positively phrased EBPAS items and generally lower for negatively phrased items (in the Divergence scale) (S1). Mean scores for the EBPAS scales (Divergence scale reversed) were all over a neutral score of 2; a higher score indicates more positive attitudes towards adopting EBP (Table 2). The Appeal subscale had the highest score followed by the Openness, Divergence (when reversed) and Requirement subscales.

**Table 2:** Comparison between EBPAS scale scores in Swedish CAMHS and USA norms^a^

Scale	Swedish		USA Norms^1^		*t*	*Df*	Cohen’s *d*
	*M*	*SD*	*M*	*SD*			
Requirements	2.70	.80	2.41	.99	6.68***	1834.63	0.31
Appeal	3.23	.54	2.91	.68	10.90***	1851.93	0.49
Openness	2.91	.60	2.76	.75	4.81***	1862.04	0.22
Divergence	1.19	.62	1.25	.70	−1.63	1796.66	−0.08
EBPAS total	2.93	.44	2.73	.49	8.52***	1878	0.40

*EBPAS* Evidence Based Practice Assessment Scale, *CAMHS* Child and Adolescent Mental Health Services. Scores range from 0 to 4

^***^p < .0001

^a^Aarons [14]

### Comparison with norms from the USA

Compared to USA norms, participants in this study had significantly higher total and subscale scores, except for the Divergence subscale (Table 3). The effect size differences were small (Requirement and Openness) or moderate (Appeal and EBPAS Total scale).

**Table 3:** Means and standard deviations for *EBPAS scale scores* by clinician characteristics

EBPAS scales	Requirements		Appeal		Openness		Divergence		EBPAS total	
	*M*	*SD*	*M*	*SD*	*M*	*SD*	*M*	*SD*	*M*	*SD*
All	2.69	0.81	3.22	0.55	2.90	0.60	1.20	0.62	2.92	0.46
Gender										
Female	2.74_a_	0.80	3.26_a_	0.55	2.92_a_	0.60	1.18_a_	0.62	2.95_a_	0.45
Male	2.43_b_	0.83	3.04_b_	0.52	2.53_a_	0.60	1.31_a_	0.60	2.76_b_	0.46
Age										
< 45	2.66_a_	0.79	3.31_a_	0.52	3.03a	0.56	1.10_b_	0.58	2.99_a_	0.41
≥ 45	2.71_a_	0.83	3.13_b_	0.56	2.79_b_	0.62	1.29a	0.65	2.85_b_	0.48
Profession										
Other^a^	2.79_b_	0.87	3.18_a_	0.57	2.83_a_	0.66	1.28_a_	0.65	2.89_a_	0.51
Nurse	2.96_b_	0.76	3.24_a_	0.54	2.81_a_	0.64	1.20_a_	0.67	2.95_a_	0.48
Social worker	2.66_a_	0.74	3.23_a_	0.54	2.88_a_	0.59	1.21_a_	0.62	2.91_a_	0.43
Psychologist	2.58_a_	0.85	3.27_a_	0.56	2.99_a_	0.58	1.17_a_	0.60	2.95_a_	0.45
Psychiatrist^b^	2.65_a_	0.77	3.11_a_	0.54	2.93_a_	0.57	1.17_a_	0.61	2.90_a_	0.42
Education										
Bachelor or lower	2.70_a_	0.83	3.22_a_	0.56	2.86_a_	0.62	1.26_a_	0.63	2.89_a_	0.47
Master or higher	2.67_a_	0.78	3.24_a_	0.54	3.00_b_	0.56	1.05_b_	0.57	2.99_b_	0.41
Experience										
< 5 years	2.71_a_	0.81	3.28_a_	0.52	3.01_a_	0.61	1.12_a_	0.57	2.99_a_	0.43
> 5 years	2.68_a_	0.81	3.18_a_	0.57	2.83_b_	0.59	1.26_b_	0.65	2.87_b_	0.46

Scale scores are mean scores provided that at least 50% of items had valid data. Means not sharing subscripts (a or b) differ significantly at *p* < 0.01

^a^Others are auxiliary nurses and others

^b^Psychiatrist include child psychiatrists, residents, and MDs without any specialist training

### Differences in attitudes due to individual and organizational factors

Table 3 presents the means and standard deviations for the total and subscale scores on the EBPAS by groups defined by clinician characteristics. Significant between-group differences are indicated by values with different subscripts. These variables as well as attitude toward diagnosis, type of service and organizational readiness was studied as predictors of attitudes by simple and multiple regression models and are presented in Table 4. All differences between groups were in general small in relation to differences between individuals, explaining about 2.0% of the variance in the unadjusted models (Table 4).

**Table 4:** Predictors of EBPAS scale scores from simple (one Predictor) vs. multiple (controlling for others) regression analyses

	Requiremen**ts**				Appeal				Openness				Divergence				Total			
	Simple		Multiple *R*^2^ = .099		Simple		Multiple *R*^2^ = .107		Simple		Multiple *R*^2^ = .115		Simple		Multiple *R*^2^ = .124		Simple		Multiple *R*^2^ = .172	
Characteristic Gender	R^2^	β	Sr Unique	β	R^2^	β	Sr Unique	β	R^2^	β	Sr Unique	β	R^2^	β	Sr Unique	β	R^2^	β	Sr Unique	β
	.021	.145***	.13	.125***	.022	.147***	.13	.122***	ns	ns		ns	.007	-.081*	**ns**	ns	.024	.155***	**.13**	.117***
Age	ns	ns		ns	.024	- .155***	-.11	-.122**	.04	- .205***	**-.12**	-.128**	.022	.148***	**.11**	.118**	.026	- .161***	**-.10**	-.109**
Educational level	ns	ns		ns	ns	ns	ns	ns	.013	.113**		ns	.025	- .157***	-.12	-.118**	.010	.098**	ns	ns
Experience	ns	ns		ns	.007	-.086*	ns	ns	.024	- .154***	-.11	-.117**	.011	.112**		ns	.017	- .129***	ns	ns
Profession	.024				ns				.013				ns				ns			
*Other*		.092*	.096	.104**		ns	ns	ns		-.091*		ns		ns		ns		ns	ns	ns
*Nurse*		.161***	.147	.157***		ns	ns	ns		-.106**		ns		ns		ns		ns	ns	ns
*Social worker*		ns		ns		ns	ns	ns		-.085*		ns		ns		ns		ns	ns	ns
*Psychiatrist*		ns		ns		ns	-.11	-.122**		ns		ns		ns		ns		ns	ns	ns
Benefit of diagnosis	.045	.211***	.209	.212***	.038	.195***	.21	.214***	.05	.228***	.22	.220***	.09	- .293***	-.27	- .278***	.108	.329***	.33	.328**
Service	ns	ns		ns	.015	.123***	.10	.098**	.013	.112*	.10	.092*	ns	ns	ns	ns	.01	.102*	.08	.073*
Org. readiness	.015	- .121***	-.11	-.107**	.006	-.074*	ns	ns	ns	ns	ns	ns	.004	ns	.007		**.011**	-.113**	-.09	-.083*

Reference groups for dummy coded variable are as follows: gender = male, age = younger, educational level = low, Experience = short, Service = non-academic clinic Sr = semi partial correlations

^***^p < .001

^**^p < .01

^*^p < .05

*Females* scored significantly higher than males on all EBPAS’s scales (Divergence scale scores are reversed) except Openness. However, when controlling for multiple comparisons, only the comparisons for Requirements, Appeal, and EBPAS total scales remained significant (Tables 3 and 4). *Younger clinicians* scored higher (lower for Divergence) than older clinicians on all scales except Requirements. *Staff with a bachelor’s degree or lower* scored significantly lower than those with a master’s degree or higher on the Openness, Divergence (when reversed), and EBPAS total scale. A significant difference between professional disciplines was observed only for the Requirement scale after controlling for multiple comparison. Nurses had higher Requirement scores than psychologists. *Those with shorter experience* in child psychiatry scored significantly higher on the Openness, lower on the Divergence, and higher on the EBPAS total scale (Table 4). Finally, *attitude towards diagnosis* correlated with attitude towards EBP adoption across all domains (Table 4).

*Respondents working at academic services* reported significantly higher Appeal scores than those working in non-academic services. *Organizational readiness for change* (ORC- short form scale) had weak and negative correlations with the Requirement and Total scales, respectively.

### Differences in attitudes when controlling for the other individual and organizational factors

Table 4 presents the results of multiple regressions to test whether sex, age, educational attainment, profession, experience, attitude to diagnosis, type of service, and organizational readiness would remain significant predictors of EBP attitudes, following the analytical plan of the original EBPAS studies (Aarons 2004, 2010), rather than the more complicated two-level analytic plan of Aarons 2012 (Aarons 2012). Semipartial correlations (also called part correlations) indicate the “unique” contribution of an IV to the DV. Specifically, the squared semipartial correlation indicates how much R^*2*^ will decrease if that variable is removed from the regression equation. The supplementary material provides a detailed description of the results regarding predictors for each attitudinal domain.

When controlling for other individual and organizational factors in the adjusted models, the results regarding *females, younger clinicians*, respondents working at *academic services*, and, not least, *attitude towards diagnosis* remained the same (Table 4)*. Experience* in child psychiatry remained significant only for Openness, *educational level* only for the Divergence scale and *organizational readiness for change* only for the Requirement scale. For *professional discipline*, compared to psychologists, the ‘others’ group, in addition to nurses, scored higher on the Requirement scale, while psychiatrists scored lower on the Appeal scale in the adjusted models.

## Discussion

This study aimed to address several knowledge and methodological gaps in the literature about EBP attitudes among CAMHS clinicians, and how clinician and organizational characteristics might relate to these attitudes. To date, this is the first study carried out in Sweden of CAMHS clinician’s EBP attitudes towards both EBP interventions and assessment, and one of the largest studies of EBP attitudes among CAMHS clinicians in any country. Overall, we found that CAMHS clinicians across Sweden and from various disciplines (*n* = 799) reported generally favourable attitudes towards EBP, with subgroup differences mainly at the clinician rather than the organizational level (discussed below). EBP adoption attitudes in this sample were similar to those assessed by the EBPAS in a Norwegian study and somewhat more positive than USA norms for EBPAS [19, 23]. The best predictor of positive EBP attitudes was holding favourable views of the utility of psychiatric diagnosis. Before discussing the implications of the current findings, we briefly highlight the findings for clinician- and organizational-level variations in EBP attitudes.

### Clinicians’ attitudes toward EBP adoption

Participants in this study reported favourable attitudes towards EBP overall and on all four EBPAS dimensions; they were ready to adopt an EBP when appealing (Appeal), they were open to new and research-based treatments (Openness), and they perceived relatively little divergence from EBP and their current practice (Divergence). Likewise, they were positive towards, but slightly less inclined to use, mandatory EBPs (Requirement). This pattern of attitudes across domains (subscales) was similar to that reported in a Norwegian study and in the USA norms study [19, 23].

### Comparison with norms from the USA

Broadly aligned with the Norwegian study, but in contrast with a Greek study, CAMHS clinicians in this study held more favourable EBP attitudes compared to their counterparts in the USA [19, 23, 31]. The positive EBP attitudes in the Swedish sample, particularly on Openness and Appeal, may have been influenced by their positive attitudes towards the specific innovation being implemented [48, 49]. A companion study with a subsample of these participants indicated a positive view of the guideline’s characteristics and a moderate relationship between guideline attributes and EBP adoption attitudes [39, 40]. Regarding lack of differences on the divergence scale, a more detailed analysis suggests that the Swedish and American samples equally valued their own judgment on how to care for their patients compared to researchers and were equally prepared to use structured interventions. However, the Swedish clinicians were a bit more sceptical than their American counterparts about the clinical utility of research-based interventions. This scepticism may stem from a perception that these interventions are not tailored to the Swedish healthcare context. Furthermore, results suggest that the Swedish sample put less emphasis on clinical experience and more emphasis on structured and standardized treatment methods compared to their American counterparts. A possible explanation is their comparatively less experience. Nevertheless, EBP attitudes such as Openness have been linked to EBP adoption, thereby suggesting a good starting point for the implementation effort [17, 36]. However, as Divergence has been linked to non-use and discontinuation of EBPs, addressing concerns about the clinical applicability of EBPs, by adapting them to better fit the Swedish CAMHS context and certain patient groups, and providing evidence of their feasibility and effectiveness might mitigate this potential barrier for EBP uptake [17, 34, 36, 50].

### Differences in attitudes due to individual and organizational factors

Taken together, our results indicate group differences between professions related to gender, age, experience, and education, and to some extent profession, service type and organizational readiness, aligning with prior studies [14, 19]. Consistent with Aarons et al. (2010), we found that female respondents generally held more favourable attitudes toward EBPs, as demonstrated by significantly higher ratings in the Requirements, Appeal, and EBPAS total scales, whereas more experienced clinicians were less open [19]. However, in our study, results regarding gender expanded to Divergence and Aaron´s findings on experience level expanded to include Requirements and Divergence. In our study, age was significantly associated with general attitudes and three out of four specific attitudes but not Requirement and educational level was associated to Divergence. This contrasts with Aarons’ study, where age was linked solely to the Requirement dimension and educational level linked to Requirements and Appeal. In the Swedish sample, nurses and the other discipline group scored higher on Requirement and psychiatrists lower on Appeal, while social workers scored higher on the EBPAS total and Openness scales, and the others group discipline group scored lower on Divergence in the U.S.A sample. A possible explanation for age having a greater impact than experience in our study is that participants had comparatively less experience. Additionally, differences in educational systems and professional roles, particularly in education and training in evidence-based methods, might result in Swedish clinicians with higher education being more autonomous and sceptical towards EBPs than their colleagues elsewhere. This could also account for the differences observed between professions. Results regarding discipline from these studies are however in contrast to results from our beforementioned study on guideline implementation, where psychiatrists held a more positive view than the other professions, notably compared to social workers, regarding guideline characteristics and their own ability to adopt the guideline [39]. These findings highlight differences in professionals’ broad and specific EBP attitudes across cultures and may be attributed to how participants from different disciplines interpret the concept of EBP or perceive the innovation attributes of a specific EBP such as a guideline, which may stem from cultural differences as well as from differences in the organization of CAMHS and the education of CAMHS clinicians. These findings indicate that implementation theory can gain by paying more attention to differences between educational systems, organizational structures and professional cultures in different national contexts, thereby developing more sensitivity towards the contingent nature of the weights and meaning of different factors influencing implementation processes and outcomes.

While no differences emerged between CAMHS (according to the ICCs), we observed associations between service characteristics and specific attitudes: clinicians in academic settings found EBP more appealing than those in non-academic settings; and organizational readiness was uniquely linked to the Requirements scale.

Our prediction models accounted for relatively small portions of the overall variance in each EBPAS scales. In that context, positive attitudes towards diagnosis were the best predictor across dimensions and models. This finding might reflect that most (if not all) evidence-based treatment protocols / care guidelines in CAMHS are diagnosis based [10]. Given the gaps in existing literature, making comparisons is challenging. EBPs are often criticized for not accommodating the complex and comorbid nature of patients in real-world settings. However, research indicates that children who receive diagnosis-congruent EBTs in community settings show better improvements [51]. This positive effect also extends to patients with comorbid disorders, compared to those not receiving EBT. Therefore, it would be interesting to investigate whether clinicians with positive attitudes toward EBPs see more value in psychiatric diagnosis due to their better treatment outcomes or if the reverse is true.

These findings, if replicated, may have implications for implementation theory. Most importantly, our results indicate that the attitude towards diagnosis plays a more significant role in shaping attitudes towards EBP adoption compared to other individual and organizational factors, although it is unclear what direction this relationship takes or its importance for EBP uptake. Additionally, our findings from previous studies suggest an interconnectedness not only of context factors, as suggested by the EPIS framework, but also between specific innovation characteristics and inner context factors like EBP adoption attitudes [52]. Furthermore, EBP adoption attitudes seem to differ less depending on professional discipline compared to the perception of specific innovation attributes, indicating that innovation attributes are not fixed but dependent on potential users’ perceptions, as suggested by Rogers, providing additional evidence for this connection [53]. Regarding implications for implementation projects, our findings suggest that implementation planners cannot rely solely on findings from other countries but need to tailor their approaches to what is being implemented based on the adopters’ needs.

### Strengths and limitations

This first replication of Aarons study investigating clinician attitudes towards the adoption of EBP benefitted from a large and representative sample of front-line CAMHS clinicians from diverse professions from across Sweden. The robust response rate of 60% exceeded commonly viewed thresholds for e-mailed surveys and our sample size was sufficient to support the statistical analyses. Regarding limitations, we were unable to obtain data about non-respondents to the survey to investigate any potential selection bias, such that attitudes could be over- or underestimated. Reflecting the nature of the CAMHS outpatient workforce in Sweden, the professional groups differed as expected in size [54]. Generally, professions with a more positive view outnumbered the more negative ones resulting in a more positive view.

These results may not generalize to all implementation efforts. Participating CAMHS applied to join the implementation program for adopting clinical guidelines on depression in young people. Their staff may be more positive towards EBP compared to clinicians at CAMHS that did not participate. The voluntary participation may also explain the lack of significant differences between CAMHS (according to ICC). However, it is worth noting that a substantial number of publicly owned and operated CAMHS participated, while several of the non-participating CAMHS were in the process of joining.

Our study did not incorporate data from the USA, caution is therefore warranted when interpreting the observed EBP attitude differences between Sweden and the USA. The findings, beyond highlighting potential cultural distinctions, can also partly stem from variations in sample characteristics, data collection methods and the 15-year time span since the establishment of USA norms.

Another limitation is the cross-sectional design, which prevents us from establishing the directionality of the observed effects. For instance, it is possible that clinicians’ attitudes toward EBP adoption influence their perceptions of the utility of psychiatric diagnosis, rather than the other way around.

The regression models predicted 10–17% of the total variances, in line with previous studies [13, 14, 19], reflecting the complex mechanism involved in creating attitudes and that additional factors than those studied contribute to a large degree to attitudes towards EBP adoption. One reason for weak results is that our study employed just a single item to assess perceived benefits of diagnosis and only nine items to gauge organizational readiness, which may decrease reliability of findings.

### Implications

Our findings suggest a promising start for implementing evidence-based practice within Swedish CAMHS, particularly from the clinicians’ perspective. Clinicians generally value and are willing to adopt EBPs, and this positivity is remarkably consistent across professional groups and services. Nevertheless, our results indicate that early EBP educational efforts might be most effective when focused on enhancing the perceived benefit of psychiatric diagnosis. However, the extent of fine-tuning required will become clearer once utilization and satisfaction data with EBP are obtained through longitudinal investigations. Similarly, further research is needed to assess the ability of EBPAS scores to predict the adoption of, fidelity to, and sustainment of EBP compared with measures designed to assess barriers and facilitators to the adoption of evidence-based guidelines and specific EBPs. Finally, additional research is needed (and planned for) to investigate the relation between EBPAS scores and clinical outcome in Swedish CAMHS and other health care settings [55].

## Conclusion

In this first, large-scale nationwide and interdisciplinary study outside the USA, CAMHS clinicians in Sweden held generally positive attitudes towards the adoption of evidence-based practice, and somewhat more so compared to USA norms. Positive attitudes towards the utility of psychiatric diagnoses emerged as the strongest predictor of positive attitudes towards EBP. The EPBAS can help identify clinician- and organizational-level factors that are important to EBP implementation efforts, and thus improving service delivery and outcomes in routine clinical care.

## Supplementary Information

Supplementary Material 1.

## Data Availability

The dataset supporting the conclusions of this article is available in the Halland Hospital Halmstad repository. The dataset used and/or analysed during current study are available from the corresponding author on reasonable request.

## Abbreviations

CAMHS: Child and adolescent mental health services
CI: Confidence interval
EBP: Evidence-based Practice
EBPAS: Evidence-based Practice Attitude Scale
EBT: Evidence -Based Treatments
EPIS: Framework Exploration, Preparation, Implementation, Sustainment framework
ICC: Intra Class correlation
NBHW: Swedish National Board of Health and Welfare
ORC: Organizational Readiness for Change Scale
StaRI: Standards of Reporting Implementation Studies
STROBE: The Strengthening the Reporting of Observational Studies in Epidemiology
USA: United States of America

## References

[1] Garland AF, Bickman L, Chorpita BF. Change what? Identifying quality improvement targets by investigating usual mental health care. Adm Policy Ment Health. 2010;37(1–2):15–26. 20177769 10.1007/s10488-010-0279-y PMC2874058

[2] Merikangas KR, He JP, Burstein M, Swendsen J, Avenevoli S, Case B, Georgiades K, Heaton L, Swanson S, Olfson M. Service utilization for lifetime mental disorders in U.S. adolescents: results of the National Comorbidity Survey-Adolescent Supplement (NCS-A). J Am Acad Child Adolesc Psychiatry. 2011;50(1):32–45. 21156268 10.1016/j.jaac.2010.10.006 PMC4408275

[3] Peters-Corbett A, Parke S, Bear H, Clarke T. Barriers and facilitators of implementation of evidence-based interventions in children and young people's mental health care - a systematic review. Child Adolesc Ment Health. 2024;29(3):242–65. 10.1111/camh.12672 37608642

[4] Chorpita BF, Daleiden EL, Ebesutani C, Young J, Becker KD, Nakamura BJ, Phillips L, Ward A, Lynch R, Trent L. Evidence-based treatments for children and adolescents: an updated review of indicators of efficacy and effectiveness. Clin Psychol Sci Pract. 2011;18(2):154–72.

[5] Williams NJ, Beidas RS. Annual Research Review: The state of implementation science in child psychology and psychiatry: a review and suggestions to advance the field. J Child Psychol Psychiatry. 2019;60(4):430–50. 30144077 10.1111/jcpp.12960 PMC6389440

[6] Sackett DL, Rosenberg WM, Gray JA, Haynes RB, Richardson WS. Evidence based medicine: what it is and what it isn’t. BMJ. 1996;312(7023):71–2. 8555924 10.1136/bmj.312.7023.71 PMC2349778

[7] Institute of Medicine Committee on Quality of Health Care in A: Crossing the Quality Chasm: A New Health System for the 21st Century. Washington (DC): National Academies Press (US), 2001.

[8] American Psychological Association. Policy statement on evidence-based practice in psychology. 2021. https://www.apa.org/practice/guidelines/evidence-based-statement.

[9] Spring B. Evidence-based practice in clinical psychology: What it is, why it matters; what you need to know. J Clin Psychol. 2007;63(7):611–31. 17551934 10.1002/jclp.20373

[10] Harvey AG, Gumport NB. Evidence-based psychological treatments for mental disorders: modifiable barriers to access and possible solutions. Behav Res Ther. 2015;68:1–12. 25768982 10.1016/j.brat.2015.02.004 PMC4395546

[11] First MB, Rebello TJ, Keeley JW, Bhargava R, Dai Y, Kulygina M, Matsumoto C, Robles R, Stona A-C, Reed GM. Do mental health professionals use diagnostic classifications the way we think they do? A global survey World Psychiatry. 2018;17(2):187–95. 29856559 10.1002/wps.20525 PMC5980454

[12] Powell BJ, Mandell DS, Hadley TR, Rubin RM, Evans AC, Hurford MO, Beidas RS. Are general and strategic measures of organizational context and leadership associated with knowledge and attitudes toward evidence-based practices in public behavioral health settings? A cross-sectional observational study. Implement Sci. 2017;12(1):64. 28499401 10.1186/s13012-017-0593-9 PMC5429548

[13] Aarons GA. Mental health provider attitudes toward adoption of evidence-based practice: the Evidence-Based Practice Attitude Scale (EBPAS). Ment Health Serv Res. 2004;6(2):61–74. 15224451 10.1023/b:mhsr.0000024351.12294.65 PMC1564126

[14] Aarons GA, Glisson C, Green PD, Hoagwood K, Kelleher KJ, Landsverk JA, Research Network on Youth Mental H, Weisz JR, Chorpita B, Gibbons R, et al. The organizational social context of mental health services and clinician attitudes toward evidence-based practice: a United States national study. Implement Sci. 2012;7:56. 22726759 10.1186/1748-5908-7-56 PMC3444886

[15] Ajzen I. The theory of planned behavior. Organ Behav Hum Decis Process. 1991;50(2):179–211.

[16] Bandura A. Self-efficacy: toward a unifying theory of behavioral change. Psychol Rev. 1977;84(2):191–215. 847061 10.1037//0033-295x.84.2.191

[17] Becker EM, Smith AM, Jensen-Doss A. Who’s using treatment manuals? A national survey of practicing therapists. Behav Res Ther. 2013;51(10):706–10. 23973815 10.1016/j.brat.2013.07.008

[18] Becker-Haimes EM, Franklin M, Bodie J, Beidas RS. Feasibility and acceptability of a toolkit to facilitate clinician use of exposure therapy for youth. Evid Based Pract Child Adolesc Ment Health. 2017;2(3–4):165–78. 30740525 10.1080/23794925.2017.1383867 PMC6368186

[19] Aarons GA, Glisson C, Hoagwood K, Kelleher K, Landsverk J, Cafri G. Psychometric properties and U.S. National norms of the Evidence-Based Practice Attitude Scale (EBPAS). Psychol Assess. 2010;22(2):356–65. 20528063 10.1037/a0019188 PMC3841109

[20] Aarons GA, McDonald EJ, Sheehan AK, Walrath-Greene CM. Confirmatory factor analysis of the Evidence-Based Practice Attitude Scale in a geographically diverse sample of community mental health providers. Adm Policy Ment Health. 2007;34(5):465–9. 17619137 10.1007/s10488-007-0127-x

[21] Dorsey CN, Mettert KD, Puspitasari AJ, Damschroder LJ, Lewis CC. A systematic review of measures of implementation players and processes: Summarizing the dearth of psychometric evidence. Implement Res Pract. 2021;2:26334895211002470. 37089997 10.1177/26334895211002474 PMC9978628

[22] Landsverk NG, Olsen NR, Brovold T. Instruments measuring evidence-based practice behavior, attitudes, and self-efficacy among healthcare professionals: a systematic review of measurement properties. Implement Sci. 2023;18(1):42. 37705031 10.1186/s13012-023-01301-3 PMC10500884

[23] Egeland KM, Ruud T, Ogden T, Lindstrom JC, Heiervang KS. Psychometric properties of the Norwegian version of the Evidence-Based Practice Attitude Scale (EBPAS): to measure implementation readiness. Health Res Policy Syst. 2016;14(1):47. 27316675 10.1186/s12961-016-0114-3 PMC4912744

[24] van Sonsbeek MA, Hutschemaekers GJ, Veerman JW, Kleinjan M, Aarons GA, Tiemens BG. Psychometric properties of the Dutch version of the Evidence-Based Practice Attitude Scale (EBPAS). Health Res Policy Syst. 2015;13:69. 26572126 10.1186/s12961-015-0058-z PMC4647622

[25] Santesson AHE, Bäckström M, Holmberg R, Perrin S, Jarbin H. Confirmatory factor analysis of the Evidence-Based Practice Attitude Scale (EBPAS) in a large and representative Swedish sample: is the use of the total scale and subscale scores justified? BMC Med Res Methodol. 2020;20(1):254. 33054717 10.1186/s12874-020-01126-4 PMC7557010

[26] Becker-Haimes EM, Okamura KH, Wolk CB, Rubin R, Evans AC, Beidas RS. Predictors of clinician use of exposure therapy in community mental health settings. J Anxiety Disord. 2017;49:88–94. 28475946 10.1016/j.janxdis.2017.04.002 PMC5501186

[27] Farahnak LR, Ehrhart MG, Torres EM, Aarons GA. The Influence of Transformational Leadership and Leader Attitudes on Subordinate Attitudes and Implementation Success. Journal of Leadersh Organ Stud. 2020;27(1):98–111.

[28] Frazier SL, Bearman SK, Garland AF, Atkins MS: Dissemination and implementation in children's mental health: Closing the research to training gap. In: Dissemination and implementation of evidence-based practices in child and adolescent mental health. edn. New York, NY, US: Oxford University Press; 2014: 98–123.

[29] Ashcraft RGP, Foster SL, Lowery AE, Henggeler SW, Chapman JE, Rowland MD. Measuring practitioner attitudes toward evidence-based treatments: a validation study. J Child Adolesc Subst Abuse. 2011;20(2):166–83.

[30] Rye M, Friborg O, Skre I. Attitudes of mental health providers towards adoption of evidence-based interventions: relationship to workplace, staff roles and social and psychological factors at work. BMC Health Serv Res. 2019;19(1):110. 30736786 10.1186/s12913-019-3933-4 PMC6368721

[31] Melas CD, Zampetakis LA, Dimopoulou A, Moustakis V. Evaluating the properties of the Evidence-Based Practice Attitude Scale (EBPAS) in health care. Psychol Assess. 2012;24(4):867–76. 22409449 10.1037/a0027445

[32] Nilsen P. Making sense of implementation theories, models and frameworks. Implement Sci. 2015;10:53. 25895742 10.1186/s13012-015-0242-0 PMC4406164

[33] Izmirian SC, Nakamura BJ. Knowledge, Attitudes, Social Desirability, and Organizational Characteristics in Youth Mental Health Services. J Behav Health Serv Res. 2016;43(4):630–47. 26645291 10.1007/s11414-015-9491-6

[34] Beidas R, Skriner L, Adams D, Wolk CB, Stewart RE, Becker-Haimes E, Williams N, Maddox B, Rubin R, Weaver S, et al. The relationship between consumer, clinician, and organizational characteristics and use of evidence-based and non-evidence-based therapy strategies in a public mental health system. Behav Res Ther. 2017;99:1–10. 28865284 10.1016/j.brat.2017.08.011 PMC5681428

[35] Becker-Haimes EM, Williams NJ, Okamura KH, Beidas RS. Interactions between clinician and organizational characteristics to predict cognitive-behavioral and psychodynamic therapy use. Adm Policy Ment Health. 2019;46(6):701–12.31346845 10.1007/s10488-019-00959-6

[36] Beidas RS, Marcus S, Aarons GA, Hoagwood KE, Schoenwald S, Evans AC, Hurford MO, Hadley T, Barg FK, Walsh LM, et al. Predictors of community therapists’ use of therapy techniques in a large public mental health system. JAMA Pediatr. 2015;169(4):374–82. 25686473 10.1001/jamapediatrics.2014.3736 PMC4420189

[37] Meza RD, Triplett NS, Woodard GS, Martin P, Khairuzzaman AN, Jamora G, Dorsey S. The relationship between first-level leadership and inner-context and implementation outcomes in behavioral health: a scoping review. Implement Sci. 2021;16(1):69. 34229706 10.1186/s13012-021-01104-4 PMC8259113

[38] Danielson M, Månsdotter A, Fransson E, Dalsgaard S, Larsson JO. Clinicians’ attitudes toward standardized assessment and diagnosis within child and adolescent psychiatry. Child Adolesc Psychiatry Ment Health. 2019;13(1):9. 30792803 10.1186/s13034-019-0269-0 PMC6371426

[39] Santesson A, Holmberg R. Bäckström M Gustafsson, P; Perrin, S; Jarbin H: Multilevel barriers to guideline implementation - a nationwide multi-professional cross- sectional study within child and adolescent psychiatry. Child Adolesc Psychiatry Ment Health. 2024;18:115. 39267088 10.1186/s13034-024-00803-2 PMC11397028

[40] Santesson AHE, Jarbin H, Holmberg R, Gustafsson P, Perrin S, Bäckström M: The Swedish version of the Barrier and Facilitator Assessment Instrument: translation, adaptation and initial psychometric testing in a large and nationwide sample. 2024.

[41] Lehman WE, Greener JM, Simpson DD. Assessing organizational readiness for change. J Subst Abuse Treat. 2002;22(4):197–209. 12072164 10.1016/s0740-5472(02)00233-7

[42] Billsten J, Fridell M, Holmberg R, Ivarsson A. Organizational Readiness for Change (ORC) test used in the implementation of assessment instruments and treatment methods in a Swedish National study. J Subst Abuse Treat. 2018;84:9–16. 29195597 10.1016/j.jsat.2017.10.004

[43] Pinnock H, Epiphaniou E, Sheikh A, Griffiths C, Eldridge S, Craig P, Taylor SJ. Developing standards for reporting implementation studies of complex interventions (StaRI): a systematic review and e-Delphi. Implement Sci. 2015;10:42. 25888928 10.1186/s13012-015-0235-z PMC4393562

[44] von Elm E, Altman DG, Egger M, Pocock SJ, Gøtzsche PC, Vandenbroucke JP. The Strengthening the Reporting of Observational Studies in Epidemiology (STROBE) statement: guidelines for reporting observational studies. Ann Intern Med. 2007;147(8):573–7. 17938396 10.7326/0003-4819-147-8-200710160-00010

[45] IBM: IBM SPSS statistics for windows In., 27 edn. Armonk, NY; 2020.

[46] Cohen J. Statistical power and analysis for behavioral sciences, 2nd. ed. New York: Lawrence Erlbaum Associates; 1988.

[47] Tabachnick BG, Fidell LS: Using multivariate statistics: Allyn & Bacon/Pearson Education; 2007.

[48] Barnett M, Brookman-Frazee L, Regan J, Saifan D, Stadnick N, Lau A. How Intervention and implementation characteristics relate to community therapists’ attitudes toward evidence-based practices: a mixed methods study. Adm Policy Ment Health. 2017;44(6):824–37. 28236076 10.1007/s10488-017-0795-0 PMC5568987

[49] Cook JM, Thompson R, Simiola V, Wiltsey Stirman S, Schnurr PP. Provider general attitudes versus specific perceptions of evidence-based psychotherapies for PTSD. Psychol Serv. 2020;17(1):46–53. 30265069 10.1037/ser0000280 PMC6437015

[50] Lau AS, Lind T, Crawley M, Rodriguez A, Smith A, Brookman-Frazee L. When Do Therapists Stop Using Evidence-Based Practices? Findings from a Mixed Method Study on System-Driven Implementation of Multiple EBPs for Children. Adm Policy Ment Health. 2020;47(2):323–37. 31720914 10.1007/s10488-019-00987-2 PMC7237196

[51] Lee P, Lang JM, Vanderploeg JJ, Marshall T. Evidence-based treatments in community mental health settings: use and congruence with children’s primary diagnosis and comorbidity. Res Child Adolesc Psychopathol. 2022;50(4):417–30. 34661782 10.1007/s10802-021-00877-y

[52] Aarons GA, Hurlburt M, Horwitz SM. Advancing a conceptual model of evidence-based practice implementation in public service sectors. Admin Policy Ment Health Ment Health Serv Res. 2011;38(1):4–23. 10.1007/s10488-010-0327-7 PMC3025110 21197565

[53] Rogers EM: Diffusion of Innovation, 3rd ed. edn. New York: London: Free Press; 1983.

[54] Uppdrag Psykisk Hälsa S: Kartläggning Barn- och ungdomspsykiatrin 2016 In. https://www.uppdragpsykiskhalsa.se/assets/uploads/2017/11/Kartla%CC%88ggning-2016-%E2%80%93-Barn-och-ungdomspsykiatri.pdf: SALAR Swedish Association of Local Authorities and Regions/ SKL Sveriges kommuner och landsting; 2017.

[55] Ekelöf K, Sæther E, Santesson A, Wilander M, Patriksson K, Hesselman S, Thies-Lagergren L, Rabe H, Andersson O. A hybrid type I, multi-center randomized controlled trial to study the implementation of a method for Sustained cord circulation And VEntilation (the SAVE-method) of late preterm and term neonates: a study protocol. BMC Pregnancy Childbirth. 2022;22(1):593. 35883044 10.1186/s12884-022-04915-5 PMC9315331

