# Supplementary material for: Clinician attitudes towards adoption of evidence-based practice: a nationwide multiprofessional cross-sectional study of child and adolescent mental health services in Sweden
Source: BMC Health Serv Res. 2024 Nov 19;24:1432. doi: 10.1186/s12913-024-11934-9 (PMC11575185; doi:10.1186/s12913-024-11934-9)
Supplement: Supplementary file 1 — Supplementary Material 1. [file 12913_2024_11934_MOESM1_ESM.pdf]

Attitudes of EBP adoption

Supplemental table S1 EBPAS scale and item analysis

EBPAS scales, item number and description		N	*M*	*SD*	Skew-ness	Kurtosis	Med-ian	IQ-range	Miss%
Requirements		779	2.70	.80	-.131	-.492	2.67	1	
11	Supervisor required	780	2.52	.95	-.235	-.354	3.0	1	2.4
12.	Agency required	779	2.61	.90	-.28	-.35	3.0	1	2.5
13.	State required	783	2.93	.87	-.54	.02	3.0	2	2.0
Appeal		782	3.23	.54	-.29	.18	3.25	.75	
9.	Intuitively appealing	783	3.10	.77	-.57	.15	3.0	1	2.0
10.	Make sense	787	3.23	.67	-.51	.26	3.0	1	1.5
14.	Colleagues happy with therapy	780	3.10	.73	-.61	.58	3.0	1	2.4
15.	Enough training	785	3.44	.68	-1.16	1.65	4.0	1	1.8
Openness		786	2.91	.60	-.23	-.49	3.00	.75	
1.	Like new therapy types	791	2.91	.73	-.29	.05	3.0	1	1.0
2.	Will follow a treatment manual	787	2.99	.82	-.70	.80	3.0	1	1.5
4.	Therapy developed by researchers	776	3.08	.73	-.54	.15	3.0	1	2.9
8.	Therapy different than usual	778	2.64	.82	-.67	-.12	3.0	1	2.6
Divergence		780	1.19	.62	.27	-.42	1.25	.75	13
3.	Know better than researchers	773	1.22	.91	-.33	-.28	1.0	1	3.3
5.	Research-based treatments not useful	776	.98	.93	-.56	-.48	1.0	1	2.9
6.	Clinical experience more important	779	1.87	.85	.05	.37	2.0	1	2.5
7.	Would not use manualized therapy	775	0.71	.91	-1.13	.58	.00	1	3.0
EBPAS Total		782	2.93	.44	-.13	-.17	2.93	.53	11

Note Likert scale 0-4, response options 0=” not at all” to 4=” to a very great extent”

Organizational readiness for change

Supplemental table 2 Means, standard deviation and internal consistency results for ORC items used

Item		N	*M*	*SD*	*r / α*
1	Our organization needs guidance in defining its mission	781	3.69	1.10	.47
2	Our organization needs guidance in evaluating staff performance	787	3.90	0.97	.36
3	There is too much friction among staff members	790	2.68	1.10	.37
4	Staff members are given broad authority in carrying out their duties*	793	2.17	0.88	.29
5	The heavy workload reduces staff effectiveness	792	4.05	1.00	.33
6	We have enough open discussions about program issues*	796	2.59	1.11	.45
7	The general attitude here is to change things that aren't working*	798	2.34	0.94	.41
8	Management decisions here are well planned*	795	3.10	1.02	.35
9	Frequent staff turnover is a problem in our organization	801	3.85	1.20	.39
	Short-ORC Total	796	3.15	.57	.71

*Positive statements are reversed. Likert scale 1-5 (1 = strongly disagree, 5 = strongly agree) *r*= Corrected item total, α= Cronbach alpha, n=751for the internal consistency statistics

Utility of diagnosis

Supplemental table 3 Means, standard deviation and internal consistency results for the item used to measure utility of diagnosis

Item	N	*M*	*SD*	skew	kurtosis
It is functional to delineate child psychiatric issues by using diagnoses	743	3.66	0.92	-.44	-.00

Likert scale 1-5 (1 = strongly disagree, 5 = strongly agree) and an extra "Not applicable (N/A)" response category
Number of missing responses= 14 (1.8%). The N/A response option (n= 43, 5.4%) was treated as missing, Total number of missing responses= 57 (7.1%)

Results suggests that participants had a rather positive view regarding the utility of diagnosis in pair with results from the Danielsson et al study.

Predictors of attitudes toward adopting EBP in the multiple regression models

The multiple regression model significantly predicted Requirement scores, R^2 =0.99, F (693, 11) = 6.90, $p <$ .001, corresponding to a small effect size (Table 4). Gender (females scoring higher), profession (psychologists scored lower than nurses and others respectively), attitude toward diagnosis (more positive toward diagnosis scoring higher), and organizational readiness (more negative about readiness scoring lower) made significant contributions in the adjusted model as shown by the squared semipartial correlations. Appeal scale scores were significantly predicted by the full model, R^2 = .107, F (695, 11) = 7.53, p <.001. In line with results from the simple regression models, educational level and clinical experience did not make significant contributions. In contrast with the simple regression models, psychiatrists scored lower than psychologists on the Appeal scale; but the unique contribution to the full model was small (1.2 % of explained variance). Gender, age, attitude towards diagnosis, and type of service gave statistically significant contributions to the explained variance of the full model, unique contributions ranged from 4.2% to 4.9%. The full regression model explained 11.5% of the variance in Openness scores, R^2 = .115, F (699, 11) = 8.24, p < .001. The only predictors that remained statistically significant were age, experience, and benefit of diagnosis (unique contributions ranged from 1.0% to 4.5%), whereas educational level and profession did not give any significant contribution when controlling for the other predictors. Divergence subscale scores were significantly predicted by the full model, R^2 =.124, F (696, 11) = 8.94, p < .001. Age, educational attainment, and attitude towards diagnosis (but not level of experience) made unique contributions to the total variance of the Divergence scores (individual contributions ranging from 1.0% to 7.0%). The full model significantly predicted total scores on the EBPAS, R^2 = .172, F (699, 11) = 13.185, p < .001, a medium sized effect size. Gender (females scoring higher), age (younger scoring higher), and attitude toward diagnosis (more positive scoring higher) made significant unique contributions in the full model,

whereas level of education or experience and perceptions about organizational readiness did not make any significant contribution when controlling for the other variables.
